# Comparative Analysis of Mine Shaft Hoisting Systems’ Brake Temperature Using Finite Element Analysis (FEA)

**DOI:** 10.3390/ma15093363

**Published:** 2022-05-07

**Authors:** Florin Dumitru Popescu, Sorin Mihai Radu, Andrei Andraș, Ildiko Brînaș, Daniela Ioana Budilică, Valentin Popescu

**Affiliations:** Department of Mechanical, Industrial and Transport Engineering, University of Petroșani, 332009 Petrosani, Romania; sorin_mihai_radu@yahoo.com (S.M.R.); andrei.andras@gmail.com (A.A.); kerteszildiko@ymail.com (I.B.); danalupulescu@yahoo.com (D.I.B.); vpv.popescu@gmail.com (V.P.)

**Keywords:** mine hoist, drum-and-shoe, disc-and-pads, brake, friction, heat, stress, COMSOL

## Abstract

This paper studies both the thermal and mechanical behavior of brake system models in the case of the emergency braking of a mine hoist model. Using a step-by-step approach inspired by studies conducted on small brake systems with high rotation speeds specific to road and rail vehicles, a comparative analysis using a computer simulation was performed for the two types of brakes of a mine hoist system. A Solidworks model was built for two configurations: the drum-and-shoe and the disc-and-pads, and it was imported to COMSOL Multiphysics, where the material properties and simulation parameters were defined. Simulations were performed for each configuration, first using a Heat transfer module in the solids to investigate the frictional heat. The results showed the locations of the hot points on the disc and on the drum, with the surface temperature reaching 97 °C on the disc and 115 to 159 °C on the drum. Next, simulations using a Structural Mechanics module were run to obtain the stress and deformation induced by the heat generated during braking. The von Mises stress of the drum-and-shoe brake occurred on the external surface of the drum and had a value of 2 × 10^8^ N/m^2^. For the disc-and-pad brake, the stress occurred towards the edges of the brake pad contact and was 4 × 10^8^ N/m^2^. Both values were under the yield stress of the passive brake element material. Regarding the deformations, for the drum-and-shoe brake, it appeared towards the outer boundary of the drum, being 0.45 mm, and for the disc-and-pad brake, it was situated at the external edge of the disc, being 0.25 mm. COMSOL Multiphysics allowed the evaluation of the thermo-mechanical behavior using noninvasive techniques since actual emergency braking testing on a working mine hoisting installation is not possible because of safety and logistic concerns.

## 1. Introduction

Ore and coal deposits that are underground can be accessed either by drift mining or vertical shafts [1], depending on the terrain and depth of the seam. In the latter case, vertical access is done by utilizing mine hoisting systems. These are mechanical machines operating in vertical shafts, typically used to transport material, ores and coal from underground to the above ground, and equipment, machinery and workers into the mine. 

**Figure 1:**
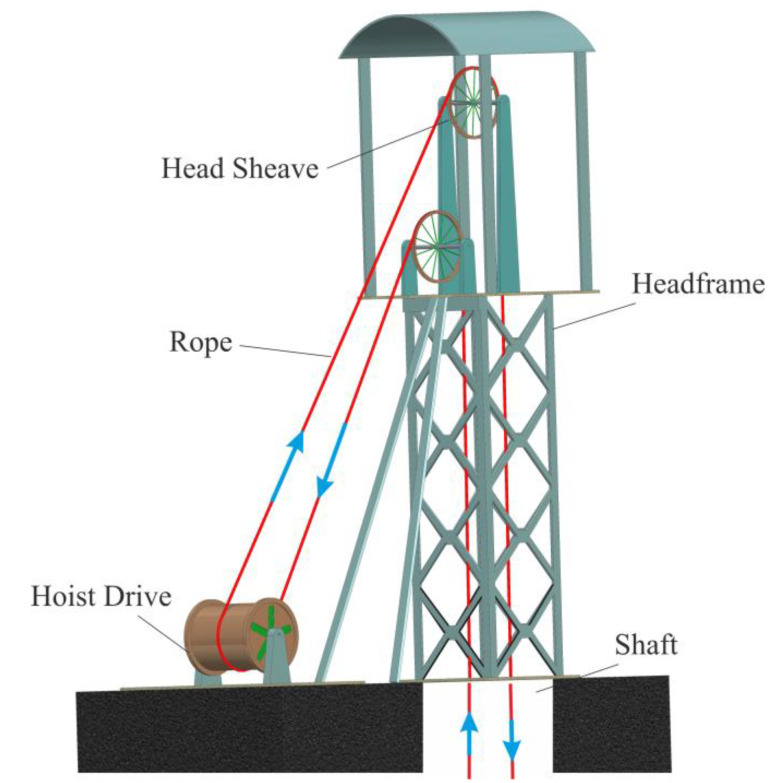
A multi-rope friction hoisting system layout.

There are three main categories of hoisting systems: drum hoists, where the hoisting cable is wound around the drum when the cage or skip is being lifted and can either be a single drum or double drum hoist; friction hoists (also called Koepe hoists), which have multiple haulage ropes that are not fixed to a drum but are passed over a friction pulley (the counterweights and tail ropes are attached to the bottom of conveyances); and Blair multi-rope hoists, which are a version of the double drum hoists where the second drum cable is used to balance the primary load [2,3,4]. The mining conditions determine the type of hoisting system used. In the case of coal mines, which usually have depths between 600 and 1200 m, the first choice is the friction hoists. Deep and very deep mines in the US, China and South Africa use drum hoists for depths of up to 2000 m or Blair hoists for depths above 2000 m. All mine hoisting systems have several major components: a hoist drive, wire ropes with conveyances (skips or cages), a headframe, sheaves and the shaft, as presented in Figure 1 for a multi-rope friction hoist.

**Figure 2:**
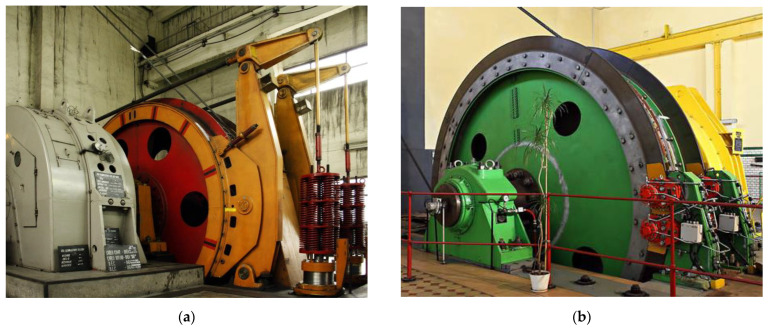
Types of brakes used in mine hoisting systems: (**a**) drum-and-shoe brake system; (**b**) disc-and-pads brake system.

The braking system selection for a mine hoist considers multiple variables such as the shaft depth, payload, levels number and drum design to maximize production and lower the hoisting cycle time. Figure 2 presents the two types of brake systems used in the case of mine hoists: the drum-and-shoe brake system (Figure 2a) and the disc-and-pads brake system (Figure 2b). Regardless of the brake system used, emergency braking, in the case of mine hoists, is used in the case of losing control over the drive motor or overspeeding in order to avoid or minimize overwinding, rope slipping or shaft impact that can lead to catastrophic failure or human life loss.

Braking systems are used in automotive, railroad and industrial applications to decelerate and stop moving vehicles safely. All braking, but especially repetitive and emergency braking, produces a temperature rise in all components of the brake systems because of the friction between the pads or shoe and a rotating disc or drum, which converts the kinetic energy into heat. A high temperature reduces performance, causes premature and abnormal wear and leads to brake fade. 

Various scholars have investigated the phenomena of frictional heat and thermal behavior using analytical, theoretical, experimental or combined approaches for various materials and setups, in drum or disc brakes. A mathematical model describing the thermal behavior of a drum-and-shoe brake system was solved in [5] using Green’s function method for impulse, unit step and trigonometric braking actions. Another analytical approach using Green’s function was made in [6], concluding that frictional heat should be dissipated to avoid a friction coefficient decrease. More analytical approaches to solve the thermal problems arising from friction during braking were accomplished in [7,8]. In the case of disc brakes, the thermal response was studied by several researchers [9,10,11] for the different materials of the disc–pad couple, theoretically and experimentally. The finite element method (FEM) is a valuable tool for investigating mechanical [12] and thermal behavior during braking by numeric simulation. The authors of [13] performed a study on the temperature field of a brake disc during hard braking using the transient thermal–structural direction coupling method and compared the results with experimental data. A new approach using FEM was developed in [14] for railway vehicles’ brake systems with simplified spatial models of friction heating. FEM and CFD were used in [15] to numerically determine the spatial temperature field in the case of ventilated discs. One of the FEM software used for transient thermal analyses is COMSOL Multiphysics, with its Heat transfer module (Heat Transfer in Solids). COMSOL was used by the authors of [16] to evaluate frictional heat and thermal expansion, in [17,18] to simulate the temperature of the disc surface as a function of time during emergency braking, for computing heat transfer through radiation, convection and conduction for disc brakes during emergency braking [19] and by researchers in [20] for the thermal and thermo-mechanical strong coupling analyses of friction pairs in the case of a pipe belt conveyor. Other studies used ANSYS in order to evaluate the thermal behavior of components during braking. In [21], a comparative thermal analysis was conducted for the different materials of three disc brakes, while study [22] evaluated the maximum temperature for brake discs by the simulation of multiple materials in different scenarios. Besides a thermal analysis, the authors of [23,24] used ANSYS to compute the deformation of structure, contact pressure and stress points in the disc–pad couple. However, this research and the results are almost entirely limited to small brake systems with high rotational speeds specific to road or rail vehicles. 

The present study also used COMSOL Multiphysics as a FEM modeling tool as it was based on a similar step-by-step approach, but the novelty is that it studied large brakes specific to mine hoisting machines. Another new feature is that a comparative simulation was performed for two types of brake setups fitted to the same mine hoist. To the best of our knowledge, this was not accomplished before in this way.

Compared to the braking systems used in vehicles, mine hoist brakes of either type are much larger in their dimensions in order to stop the huge loads transported, to have a slower rotation speed and to complete fewer rotations during a complete stop, thus reaching lower temperatures. However, in emergency braking, they must stop the conveyances as soon as possible with a deceleration under 4.5 m/s^2^ to prevent the rope from slipping on the friction wheel [25]. 

The proposed investigation of the behavior of the mine hoist brake system during emergency braking, from the point of view of the temperatures, deformations and stresses of the friction parts of the brake system, is important. The goal of such studies is to reduce the operational and maintenance costs and to increase the performance, reliability and transport capacity of mine hoists. Several authors have also studied topics regarding the braking of mine hoists. Experimental measurements of both stress and temperature were carried out in [26], but for the friction lining of the wheel. The stress and temperature distribution on the components of a mine hoist brake as a function of the initial speed and deceleration were investigated in [27]. Comparisons of experimental data and a simulation were conducted in [28], proving the validity of the 3D transient temperature field model for the hoist brake shoe only. The authors of [29] proposed new solutions to increase the reliability of the mine hoist brakes by comparing numeric simulation results to experimental measurements. A finite element model using thermo-mechanical coupling in the case of the transient thermal stress field was validated experimentally on a laboratory stand in [30]. The authors of [31] proved mine hoist brake overheating as a cause of failure, and the influence of the brake components’ surface temperature on the friction material tribological behavior during emergency stops was investigated in [32,33,34]. The influence of the maximum hoisting speed and acceleration on temperature and stress during the emergency braking of mine hoists was studied in [35], with the localization of von Mises stress and peak temperature on the median friction radius of the shoe–disc couple [36] and the concentration of the heat energy on the surface of the brake shoe concluded by the authors of [37]. The temperature distribution during braking for different positioning and numbers of pads was investigated in [38] to find an optimal arrangement. All these studies used different approaches as compared to the present paper, where a comparative analysis was conducted. To model the mine hoists widely used in Romanian mines, the present brake model was built in two constructive configurations. Under the condition of maximum deceleration without rope slippage, two simulations were run (one for each configuration), first using the Heat transfer module followed by the mechanical analyses of stress and deformation induced by the heating of the brake system elements. 

## 2. Problem Statement

**Figure 3:**
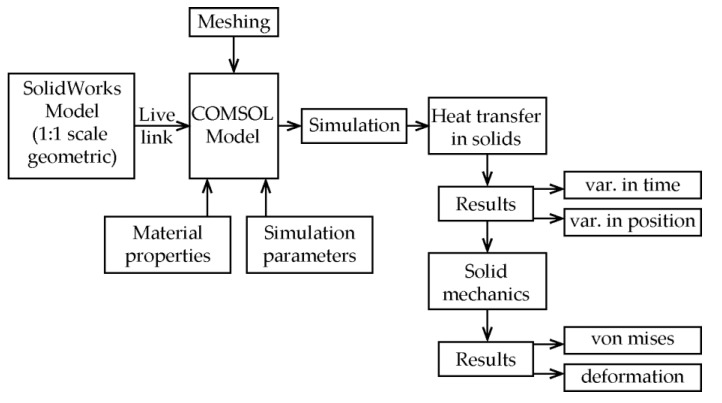
Workflow of the computer simulation.

The present study aimed to simulate the thermo-mechanical behavior of the brake system in the case of the emergency braking of a mine hoist virtual model in two configurations, the drum-and-shoe and the disc-and-pads, respectively, for the highest deceleration that meets the rope no-slip condition on the drive wheel. Since actual emergency braking testing in situ on working mine hoisting installation is not possible due to safety regulations and logistic concerns, performing this type of study using noninvasive techniques such as computer simulations is preferred. This research is based on a model developed by the same author team with published results [39,40] and it represents an improvement and extension of past research. The workflow of the computer simulation procedure undertaken is illustrated in Figure 3.

**Figure 4:**
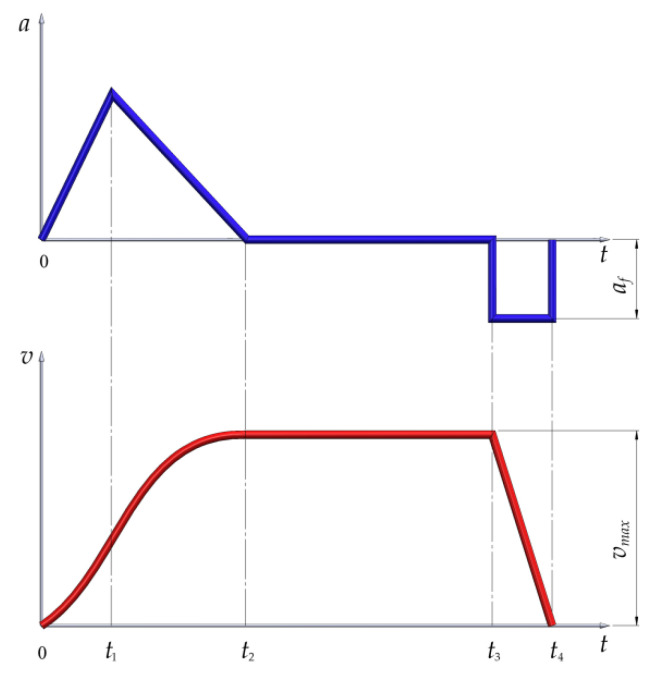
Variation of speed (*v*) and acceleration (*a*) for a single transport cycle.

It is known that the friction of surfaces during braking transforms mechanical energy to heat. The maximum temperature reached during the braking process must be under the threshold that affects the coefficient of friction, causing an abnormal wear of brake parts and a decrease in the braking performance. Mine hoists operate in transport cycles that tachograms can graphically represent. The variations in speed and acceleration for a single transport cycle is shown in Figure 4, where certain time intervals correspond to various actions: the acceleration of the conveyances from 0 to *t*_2_, the constant speed hoisting from *t*_2_ to *t*_3_ and the emergency braking under constant acceleration from *t*_3_ to *t*_4_.

**Figure 5:**
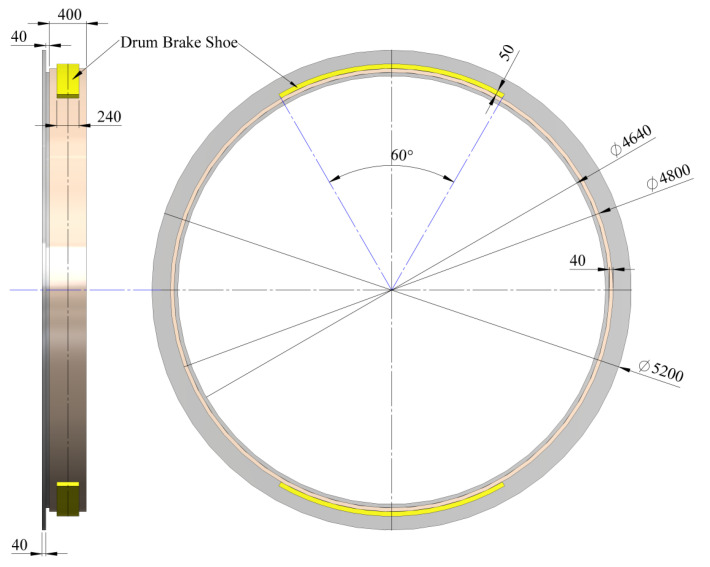
Drum-and-shoe configuration with drum, shoes and contact surface positioning and dimensions. (Unit: mm).

**Figure 6:**
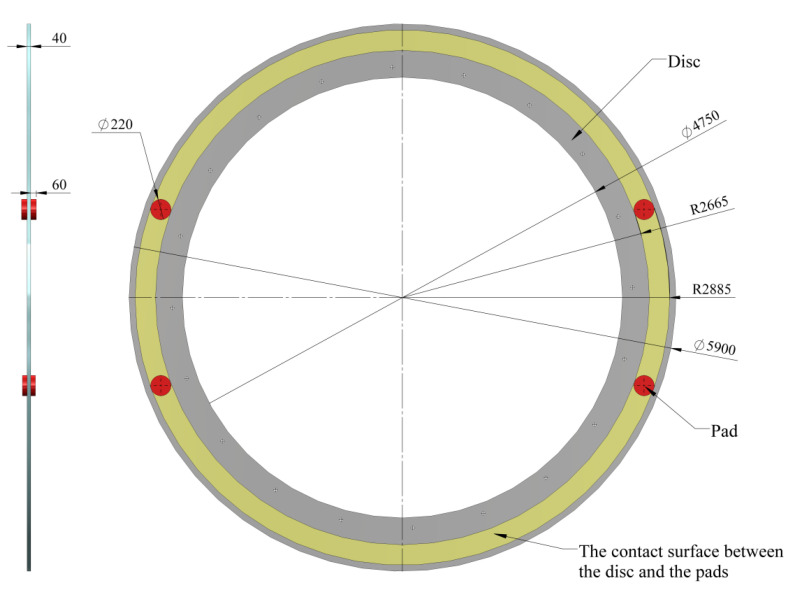
Disc-and-pads configuration with disc, pads and contact surface positioning and dimensions. (Unit: mm.

Next, the model of the mine hoist brake system is presented in both the drum-and-shoe configuration (Figure 5) and the disc-and-pads configuration (Figure 6), along with the real dimensions of the system, the positioning of the shoe in relation to the drum and the pads in relation to the disc, as well as the contact surfaces of the brake couples in both constructive setups.

In order to perform simulations of the thermal behavior of the braking systems, it is assumed that emergency braking happens when the mine hoist operates at its maximum speed; thus, the total kinetic energy can be written as:

(1) $$E_{K}{}_{max}=\frac{\Sigma m_{R}\cdot v_{max}^{2}}{2}=\frac{k\cdot P_{n}\cdot v_{max}^{2}}{2}$$

with $\Sigma m_{R}$ being the reduced mass of the hoist moving parts. Based on [3,4], this mass depends on the nominal hoisted payload *P_n_* in kilograms and a coefficient *k* depending on the conveyances content (the transport of waste rock, coal, ores, etc.), with values between 1 and 20.

Assuming that, during emergency braking, the deceleration is constant, the stopping time of the mine hoist, during which the kinetic energy is transformed to heat, can be expressed as:

(2) $$T_{STOP}=\frac{v_{max}}{a_{stop}}$$

and by deriving the kinetic energy in relation to the stopping time, the braking power is obtained:

(3) $$W_{B}=\frac{dE_{Kmax}}{dT_{STOP}}$$

The brake system has two discs or drums (one on each side), and according to [41,42], only a part of the braking power gets converted to heat on the braking parts. Thus, the heat-generating power has the form:

(4) $$W_{FR}=\frac{W_{B}}{2}\cdot 0.88$$

The actual heating of the surface of the rotating element of the brake depends on the ratio between the amount of heat at the friction of the contact surfaces (which is produced by the kinetic energy transformed to thermal energy) and the amount of heat that gets dissipated.

## 3. Heat Transfer Theoretical Principles

**Figure 7:**
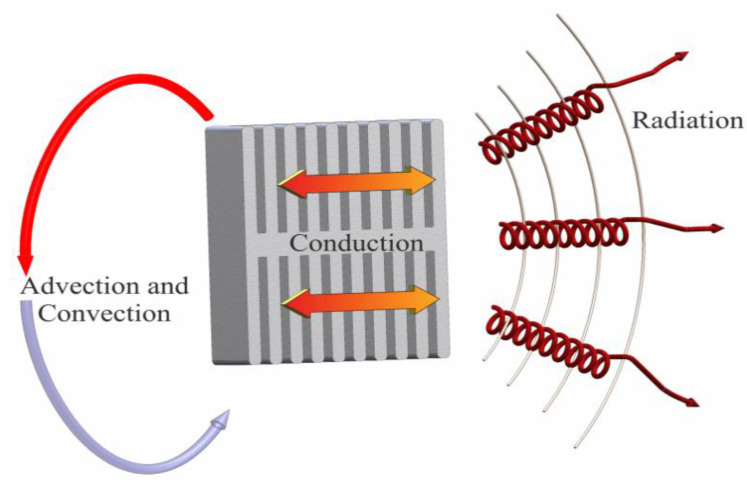
Representation of the three types of heat transfer.

When a temperature difference exists between two media or objects, heat transfer occurs. The maximum temperature reached by the braking system of the mine hoist depends on the frictional heat developed and the transferred part of that heat. Heat can be transferred in three ways: by conduction, convection or radiation, as shown in Figure 7. 

### 3.1. Heat Transfer by Conduction

Thermal conduction determines the heat flow within a body in order to equalize the differences between the internal energy, both kinetic and potential, of molecules, electrons and atoms that collide and vibrate. It is influenced by the material involved. If the material has a gapless and regular structure (such as with metals), the energy and thus the temperature equalization is done very quickly. If the material presents discontinuities and gaps (such as with refractory ceramics), the temperature equalization is slower.

**Figure 8:**
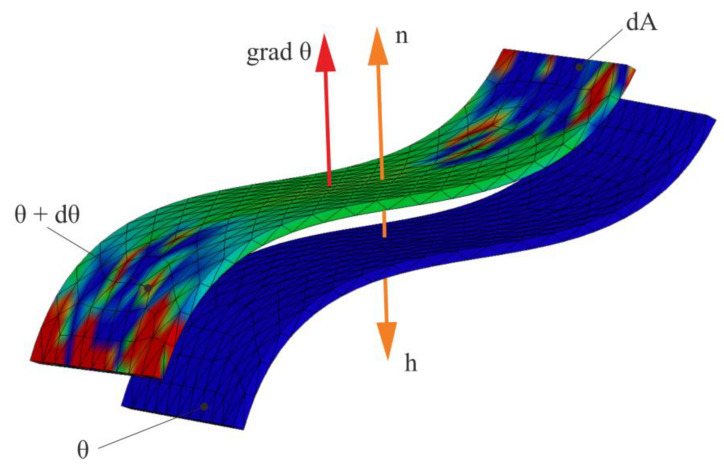
Heat transfer by thermal conduction.

The process of heat transfer through thermal conduction is presented in Figure 8. The vector $\bar{h}$ of the specific heat flux has the opposite direction to the unit vector $\hat{n}$, which is oriented in the direction of the increasing temperatures. Heat transfer occurs from higher temperature surfaces (θ + *d*θ) to lower temperature surfaces (θ).

Conductive heat transfer is governed by Fourier’s law:

(5) $$h=-n\frac{dq}{dt\cdot dA}=-\lambda grad\theta$$

where *h* is the specific heat flux (W/m^2^), $\hat{n}$ is the unit vector normal to the transmissive surface, *dq* is the elementary heat (Ws), *t* is the current time (s), *dA* is the elementary transmission area (m^2^), λ is the conductivity by conduction (W/m·grd) and θ is the temperature. 

Based on Equation (5), the power transmitted locally through the closed surface Σ boundary of volume *V* can be expressed as:

(6) $$\frac{dq}{dt}=-\oint_{\Sigma }(\lambda grad\theta )\cdot dA$$

or as:

(7) $$\frac{dq}{dt}=-\int_{V\Sigma }div(\lambda grad\theta )dV$$

according to the theorem of Gauss–Ostrogradsky.

In the case of solid bodies with *V* volume that are sources of heat transferred by conduction only, parts of this heat are stored and transferred, respectively, by conduction:

(8) $$\int_{V}p_{u}dVdt=\int_{V}c_{u}\frac{\delta \theta }{\delta t}dtdV-\int_{V}div(\lambda grad\theta )dtdV$$

with *p_u_* being the power per unit volume (W/m^3^), *c_u_* being the volume-specific heat (Ws/m^3^·grd), θ being the temperature and *t* being the current time (s).

If we assume λ is constant, then from (8), the Laplace differential equation of heat transmission in bodies with heat sources can be written as:

(9) $$\frac{\delta \theta }{\delta t}=\frac{p_{1}}{c_{1}}+\frac{\lambda }{c_{1}}\Delta \theta$$

### 3.2. Heat Transfer by Radiation

If a body is heated to a temperature *T*, it transfers heat by radiation to neighboring bodies with lower temperatures, with the specific radiated heat flux defined by the Stefan–Boltzmann law as:

(10) $$P_{1r}=\sigma \cdot e_{t}(T_{1}^{4}-T_{2}^{4})$$

where *P*_1*r*_ is the specific radiated heat flux (in W/m^2^), σ is the Stefan–Boltzmann constant ($5.67\times 10^{-8}\;W/m^{2}\cdot K^{4}$), *T*_1_ is the radiating body surface temperature, *T*_2_ is the ambient temperature and *e_t_* is the total emissivity, which depends on the nature of the body and the processing and treatment of its surface.

For further numeric calculations, Equation (10) can be written:

(11) $$P_{1r}=5.7\cdot e_{t}\left[{\left(\frac{T_{1}}{100}\right)}^{4}-{\left(\frac{T_{2}}{100}\right)}^{4}\right]\;\;\;\;\;\;W/m^{2}$$

Since $T_{1}=T_{2}+\Delta T$, Equation (10) can be written as:

(12) $$P_{1r}=4\;\sigma \;e_{t}T_{2}^{3}\left(\Delta T+\frac{6\Delta T^{2}}{4T_{2}}+\frac{\Delta T^{3}}{T_{2}^{2}}+\frac{\Delta T^{4}}{4T_{2}^{3}}\right)\;\;\;\;\;W/m^{2}$$

The content of the parentheses in Equation (12) can be simplified, with errors under 20% for Δ*T* between 3–200 °C, as:

(13) $$P_{1r}=2.65\sigma e_{t}T_{2}^{3}\Delta T^{1.2}$$

### 3.3. Heat Transfer to Fluids

The transfer of heat from solids to fluids (liquids or gases) is realized by radiation and convection. Convection implies a heat transfer by the bulk motion of fluid molecules. Convection supposes a heat transfer from heated solid-to-fluid molecules, and between the contacting fluid molecules by conduction. The motion of the molecules results from a local excess of pressure and the reduction in a specific mass caused by the fluid heating in the presence of gravitational, inertia and viscous friction forces.

Newton’s equation expresses the solid-to-fluid transferred power *P*:

(14) $$P=\alpha A(T_{1}-T_{2})=\alpha A\Delta T$$

with α being the total transmissivity (in W/m^2^·grd), *A* being the area of transmission (in m^2^), *T*_1_ being the mean temperature of the solid, *T*_2_ being the temperature of the ambient temperature and Δ*T* being the temperature difference.

The total transmissivity α has a radiation component and a convection component, so its value is dependent on the separation area treatment, the temperature difference between the media and the temperature of the ambient. Based on (14), the direction of the heat transfer between the solids and media is determined; thus, the local transmissivity can be written as:

(15) $$\alpha =\frac{dP}{dA\cdot \Delta T}=\frac{P_{1}}{\Delta T}$$

with the specific transferred power being *P*_1_ (in W/m^2^).

### 3.4. Natural Convection

The Nusselt number is a dimensionless number (invariant) used to estimate the ratio of the convected thermal energy, which can be written as:

(16) $$Nu=\frac{\alpha_{c}d_{c}}{\lambda }$$

where α*_c_* is the convective heat transfer coefficient (in W/m^2^·grd), *d_c_* is the dimension (in m) and λ is the thermal conductivity of the fluid (in W/m·grd).

The Nusselt number can be expressed as a function of other invariants, as:

(17) Nu=f(Gr, Pr)

with *Gr* being the Grashof number and *Pr* the Prandtl number, expressed as:

$$\begin{array}{l} Gr=\frac{\rho^{2}d_{c}^{3}\beta g\Delta T}{\mu^{2}},\;\mathrm{and}\;\\ Pr=\frac{\mu c_{p}}{\lambda } \end{array}$$

Here, ρ is the fluid density (kg/m^3^), β is the expansion coefficient of the fluid at a constant pressure (≈1/T), μ is the Poiseuille dynamic viscosity (N·s/m^2^), *g* is the gravitational acceleration and *c_p_* is the specific heat mass of the fluid at a constant pressure (Ws/kg·grd).

In practice, (17) can be simplified and expressed as:

(18) $$Nu=0.9{\left(Gr\cdot \;Pr\right)}^{0.2}$$

The specific convective heat flux based on (18) becomes:

(19) $$P_{1c}=\alpha_{c}\Delta T=0.9{\left(\frac{\lambda^{4}c_{p}\rho^{2}\beta g}{d_{c}^{2}\mu }\right)}^{0.2}\Delta T^{1.2}\;\;\;\;\;W/m^{2}$$

thus, according to (16), the convective heat coefficient is:

(20) $$\alpha_{c}=\frac{Nu\lambda }{d_{c}}$$

### 3.5. Global Specific Power and Transmissivity

The global transmitted specific power is the sum of the powers transmitted by radiation and convection:

$$P_{1}=P_{1r}+P_{1c}$$

Thus, from (13) and (19), the global power can be expressed as:

(21) $$P_{1}=\left[2.65Ke_{t}T_{2}^{3}+0.9{\left(\frac{\lambda^{4}c_{p}\rho^{2}\beta g}{d_{c}^{2}\mu }\right)}^{0.2}\right]\Delta T^{1.2}\;\;\;\;\;W/m^{2}$$

By dividing (21) by Δ*T,* the global transmissivity is obtained as:

(22) $$\alpha =\left[2.65Ke_{t}T_{2}^{3}+0.9{\left(\frac{\lambda^{4}c_{p}\rho^{2}\beta g}{d_{c}^{2}\mu }\right)}^{0.2}\right]\Delta T^{0.2}\;\;\;\;\;W/m^{2}\cdot \mathrm{grd}$$

The Poiseuille dynamic viscosity of the air is expressed as:

(23) $$\mu =\mu_{0}{\left(\frac{\theta +273}{273}\right)}^{0.76}\;\;\;\;\;\frac{N\cdot s}{m^{2}}\;$$

where $\mu_{0}=17.19\times 10^{-6}\;N\cdot s/m^{2}$ for the air temperature θ °C. 

Additionally, for Equation (22), temperature corrections were made for the fluid density ρ and the thermal conductivity λ.

### 3.6. The Case of Solid Media Limited by Parallel Planes

In a permanent regime, the temperature field resulting from the integration of the differential Equation (9), with the terms $\frac{\delta \theta }{\delta t}$ and $\frac{p_{1}}{c_{1}}$ cancelled, can be expressed as:

(24) Δθ=0

**Figure 9:**
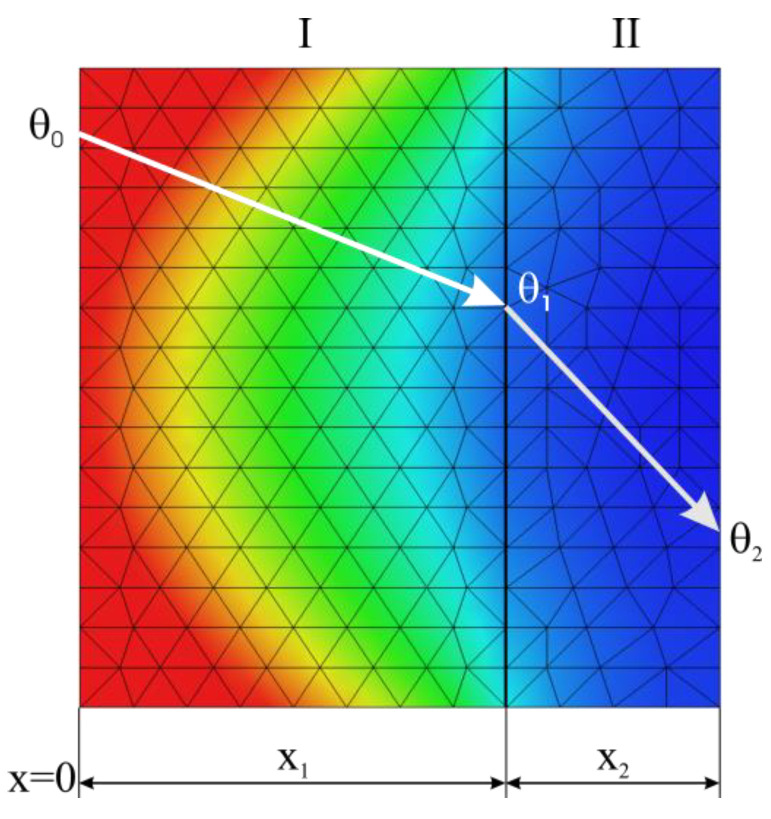
Parallel plane walls.

In this case, if it is assumed that heat is transferred in one direction only, as shown in Figure 9 where two adjacent but different media are represented, Equation (24) becomes:

(25) $$d^{2}\theta /dx^{2}=0$$

and by integration, it results in:

(26) θ=Ax+B

Here, the integration constants are determined from the boundary conditions. 

Based on Figure 9, notations of the overtemperatures are θ_0_ at *x* = 0, θ_1_ at *x* = *x*_1_ and θ_2_ at *x* = *x*_2_.

These can be written as follows:

- for $0<x<x_{1}:\theta =\theta_{0}-\frac{\theta_{0}-\theta_{1}}{x_{1}}x$;
- for $x_{1}<x_{2}:\theta =\theta_{1}-\frac{\theta_{1}-\theta_{2}}{x_{2}}x$.

For a given number *n* of solid, parallel and adjacent media, the overall conductivity can be determined from the condition of equal transmitted powers, written according to Fourier’s law:

(27) $$\frac{dq}{dt}=\lambda_{1}\frac{\theta_{0}-\theta_{1}}{x_{1}}A=\ldots =\lambda_{n}\frac{\theta_{n-1}-\theta_{n}}{x_{n}}A=\lambda_{g}\frac{\theta_{0}-\theta_{n}}{\sum_{i=1}^{n}X_{i}}A$$

where *A* is the constant area of heat transfer, λ*_g_* is the global conductivity and λ_1_…λ*_n_* are the conductivities of media 1 to *n*.

**Figure 10:**
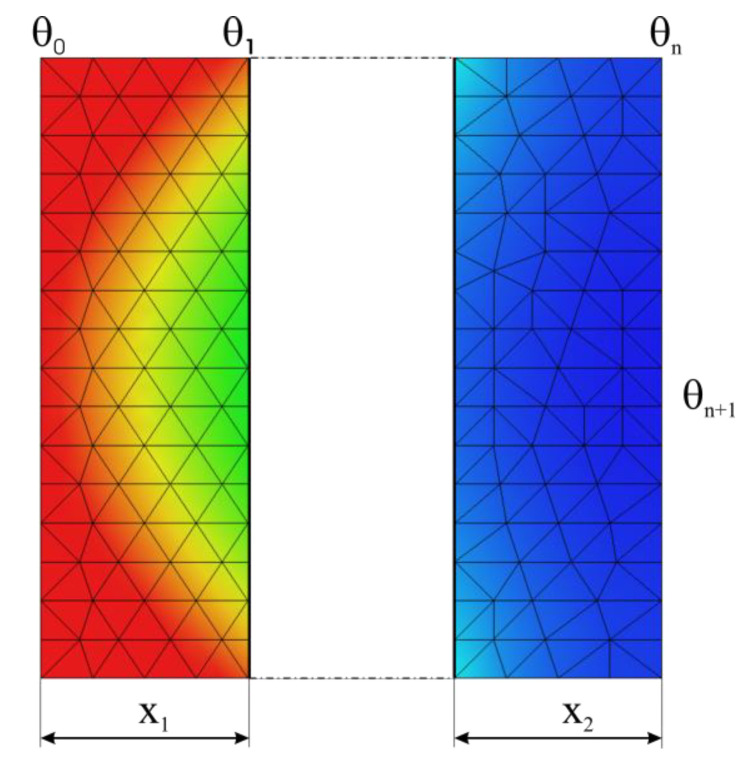
Regarding the global transmissivity α*_g_*.

By solving the system of Equation (27), with the notations in Figure 10, we obtain:

(28) $$\lambda_{g}=\frac{\sum_{i=1}^{n}X_{i}}{\sum_{i=1}^{n}\frac{x_{i}}{\lambda_{i}}}.$$

When the last solid medium transfers heat to a fluid medium, as shown in Figure 10, the global transmissivity α*_g_* is obtained from the equation of the transferred power conservation:

(29) $$\frac{dq}{dt}=\alpha_{g}A\left(\theta_{0}-\theta_{n+1}\right)=\alpha A\left(\theta_{n}-\theta_{n+1}\right)=\frac{\lambda_{g}}{\sum_{i=1}^{n}X_{i}}A\left(\theta_{0}-\theta_{n}\right)$$

where θ*_n_*_+1_ is the temperature of the fluid medium.

Thus, the global transmissivity is expressed as:

(30) $$\alpha_{g}=\frac{1}{\frac{1}{\alpha }+\sum_{i=1}^{n}\frac{x_{i}}{\lambda_{i}}}.$$

## 4. Model of the Mine Hoist Brake System

**Figure 11:**
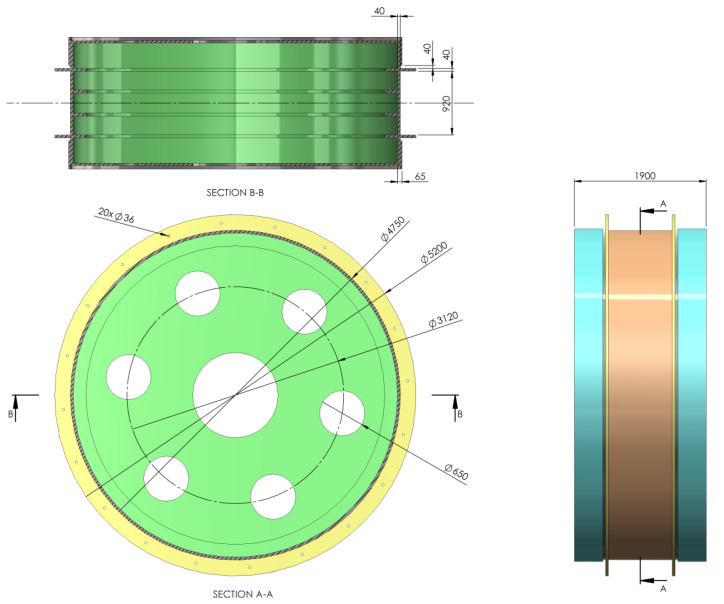
Drive wheel and drum-and-shoe brake system model for the MK5x2 hoist. (unit: mm).

**Figure 12:**
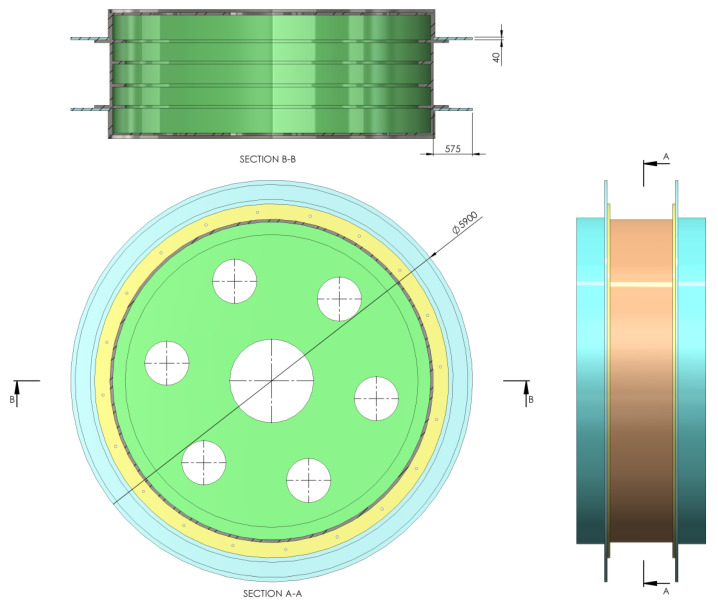
Drive wheel and disc-and-pads brake system model for the MK5x2 hoist. (unit: mm).

In order to simulate the braking process and analyze the thermal and mechanical behavior, first, the model of the drive wheel for the MK5x2 mine hoist was created at a true scale, using Solidworks. Based on the drive wheel model, both types of brake systems were then created using the same CAD software, so that the two models were finally obtained, one for the drum-and-shoe brake system (Figure 11) and one for the disc-and-pads brake system (Figure 12). It is worth mentioning that this hoist model was chosen as it is the most widespread in Romanian mines, in both the classic drum brake version as well as the modernized disc brake version in a few mine shafts. 

Regardless of the brake type used, the elements of the braking system were placed on both sides of the drive wheel, and all simulations were conducted considering a uniform pressure distribution between the couple (disc–pad and drum–shoe, respectively). The CAD models developed in Solidworks for each brake type were imported to COMSOL for the thermal (*Heat Transfer in Solids*) and mechanical (*solid mechanics*) simulations, with the material and properties of the disc, pad, drum, shoe and actual drive wheel as shown in Table 1.

**Figure 13:**
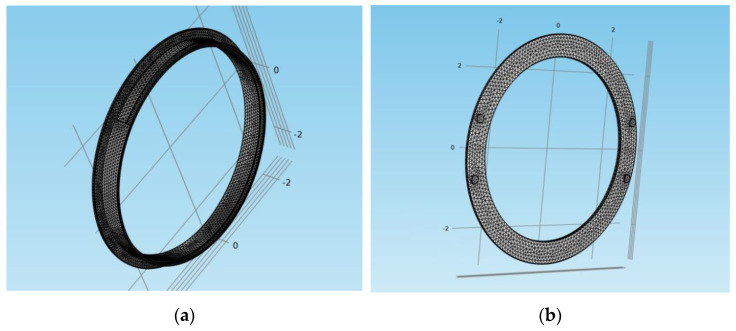
The mesh of COMSOL model: (**a**) drum-and-shoe brake system; (**b**) disc-and-pads brake system.

The finite element mesh for both models is shown in Figure 13. It was of a physics-controlled type, with the geometry constructed using tetrahedral, triangular, edge and vertex elements. The elements size was set to fine, with 46,097 elements for the drum-and-shoe model and 17,511 elements for the disc-and-pads model.

For the analyzed hoisting machine, according to its technical documentation, the maximum transport speed value was *v_max_* = 14 m/s and the payload was *P_n_* = 13,000 kg. In the case of this type of mine hoisting machine, some of its components had a fixed mass (such as the drive motor, drive wheel, rope sheaves and conveyances), while other components had a mass depending on the hoisting depth (the vertical ropes and tail ropes). Thus, in order to calculate the maximum kinetic energy, we adopted the maximum value of *k* = 20 for the coefficient *k* used for the conveyances content in Equation (1). Based on the adopted values, the calculated value of the maximum kinetic energy was $E_{c\max}=25,480,000\;J\approx 25.5\;\mathrm{MJ}$.

**Figure 14:**
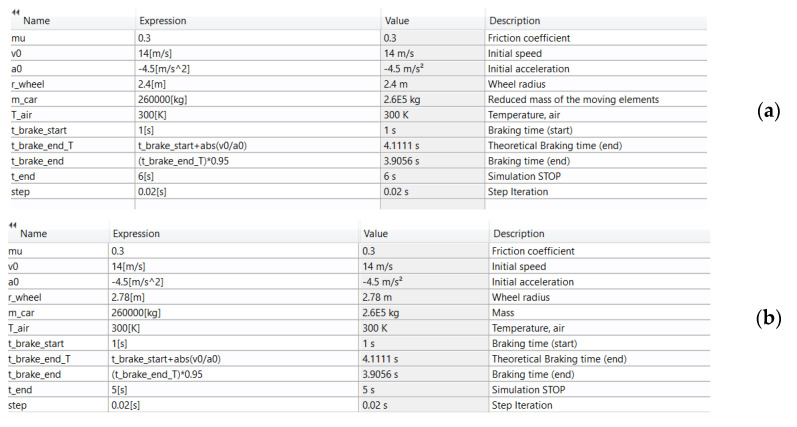
Definition of initial simulation parameters in COMSOL: (**a**) drum-and-shoe brake system; (**b**) disc-and-pads brake system.

The deceleration was considered constant during the emergency brake. The upper limit value was 4.5 m/s^2^ in order to meet the ropes no-slip condition on the drive wheel [3,4]. This value of deceleration and all other initial simulation data were defined in COMSOL using *Global definition > Parameters*, as shown in Figure 14 for both models.

**Figure 15:**
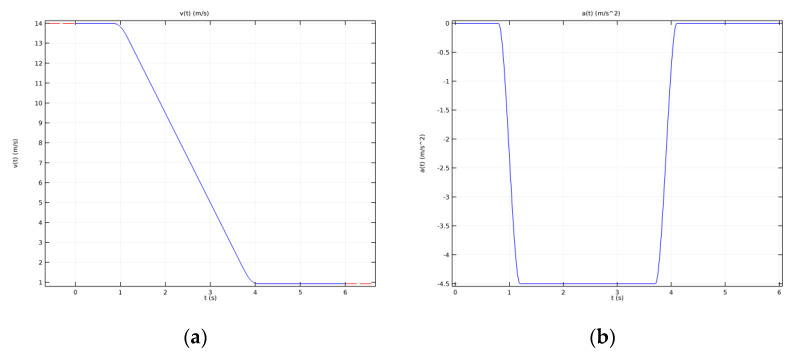
Tachogram of (**a**) speed and (**b**) deceleration during emergency braking.

Based on the above-defined initial speed, the deceleration was calculated as a derivative of the speed, and their tachogram during the 6 s emergency braking is shown in Figure 15.

Using *Global definition > Parameters*, all the faces of the disc, pads, drum, shoes, and the friction of the contact surface between the disc and pads as well as the drum and shoes were defined as being of the type *Geometric entity level: Boundary*. The external surfaces for both brake types were defined as *Geometric entity level: Domain*. At the same time, the *Component coupling > Integration* type was set between the friction of the contact surfaces previously defined and the air and the external surfaces of the components, for both brake models.

Using *Component > Heat Transfer in Solids* for both brake systems, the *Initial Values* for the temperature were set to the *Parameters T_air* value. For the *Heat Flux* in both models, first, all the boundary surfaces were selected and the *Convective heat flux option* was ticked. The values and parameters were set up according to Table 2.

The characteristics, options and values for the *Thermal Contact* of the surfaces were the same for both braking systems and are shown in Table 3.

The *Diffuse surface* was defined for the drum, shoes, disc and pads. In their case, the *Ambient temperature* was set to the value of the T_air parameter, and the *Surface Emissivity* ε value was the one in Table 1.

The form of the Global Equations corresponding to each of the two brake systems is shown in Table 4. Energy (J) and Power (W) were selected as the *dependent variable quantity*.

In *Component > Solid Mechanics*, in the case of both brake systems, for the Linear Elastic Material, the analysis was Time Dependent with the options set as in Table 5. Under the *Thermal Expansion* mode, *All domains* was selected.

The Fixed Constraint for the drum-and-shoe brake systems was the external cylindric surface of the drive wheel, and for the disc-and-pads brake system, it was its inner cylindrical edge.

## 5. Results and Discussion

### 5.1. Discussion of Thermal Behavior during Emergency Braking

After running the thermal studies for each brake model, the temperature of all the elements (the drum, shoe, disc and pad) was variable in both position and time. 

**Figure 16:**
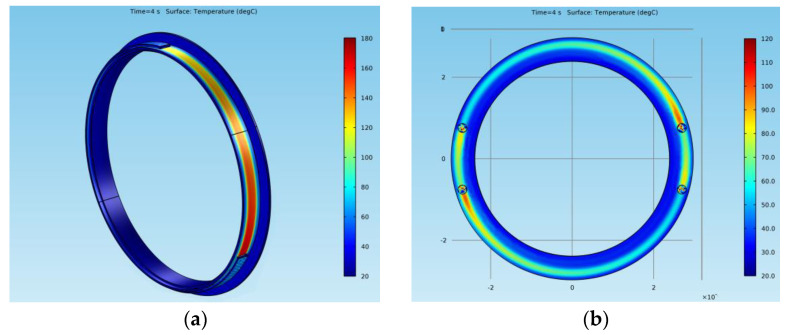
Surface temperature at *t* = 4 s for (**a**) drum-and-shoe brake system; (**b**) disc-and-pads brake system.

**Figure 17:**
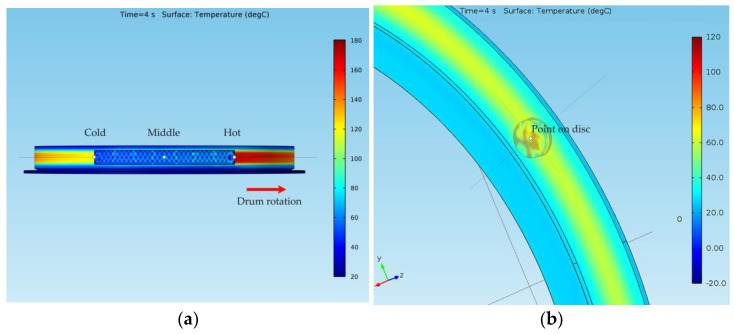
Points for surface temperature representation of the (**a**) drum-and-shoe brake system; (**b**) disc-and-pads brake system.

Figure 16 shows the temperatures of all the surfaces (*Results > Temperature (ht) > Surfaces*) for the elements of both models at a time *t* = 4 s. For the drum-and-shoe system, three points were considered, all of them situated on the surface of the drum, as shown in Figure 17a, while for the disc-and-pads system, the temperature was considered for a single point situated on the disc at the median diameter, as shown in Figure 17b.

In the case of the disc-and-pads system, the hot point was situated on the disc under the pad, while in the case of the drum-and-shoe system, the hot spot was located on the drum at the exit point from under the shoe. For both types of brake systems, it was visible that the surface temperature decreased along the rotational trajectory of the pad and shoe, respectively. Additionally, it can be observed that the temperature of the drum-and-shoe brake system was higher than the temperature of the disc-and-pads system, as the cold point of the drum had a larger temperature than the hot point of the disc.

**Figure 18:**
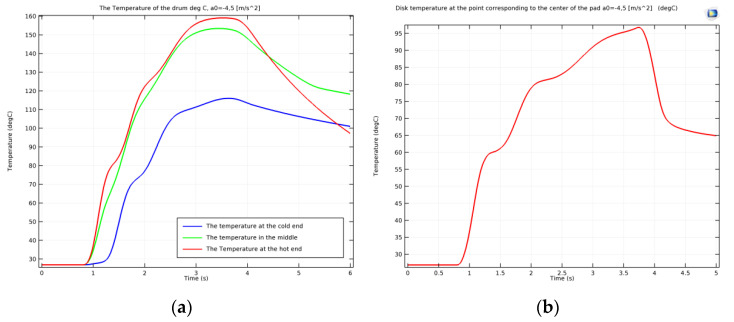
Variation in time of the surface temperature of the (**a**) drum and (**b**) disc during emergency braking.

As mentioned, the surface temperature also varied in time. In order to highlight the thermal regime for each of the systems during emergency braking, the temperature variation function of time was plotted. Figure 18a shows the variation in time of the temperature of the drum surface in the three points defined, while Figure 18b shows the variation in time of the temperature of the disc surface in the hot point under the pad.

**Figure 19:**
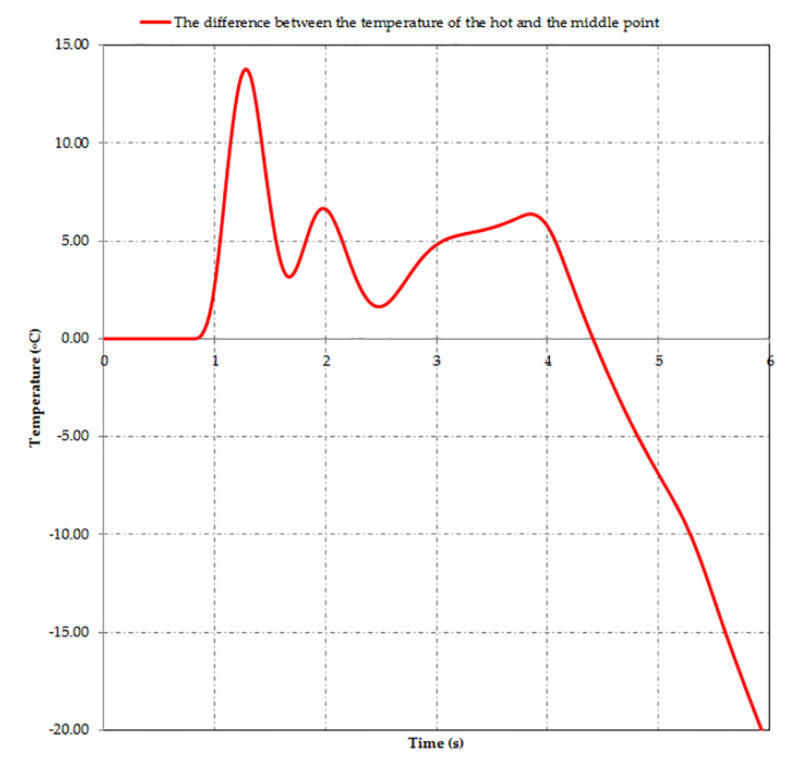
Difference between the temperature of the hot and middle point of the drum during braking.

From Figure 18, one can see that, as mentioned, the hot point of the disc had a lower temperature than the cold point of the drum, with the temperature of the disc reaching approximately 97 °C, while the drum surface presented different temperatures for the cold, medium and hot points, with values of 115, 152 and 159 °C. All the points analyzed reached their maximal temperature at the stopping moment. The graphs also show a non-uniform increase in temperature until stopping, related to the moment the investigated points on the drum and disc passed under the shoe or pad during rotation. It is visible that after *t* = 4 s (when the rotation stopped due to braking), there was a uniform cooling process in all cases. Figure 18a also shows that although during braking the temperature variation diagram of the middle point of the drum is between that of the hot and cold points, after stopping, the variation graph of the temperature of this point exceeds that of the hot point. This is explained by the middle point position of the drum under the shoe, which no longer allowed its cooling by the radiation in the air. The difference between the temperatures of the middle and hot points is shown in Figure 19.

After the emergency braking stopped the conveyances in a random position in the shaft, the active elements of the braking system (the shoes or pads) remained in contact with the passive elements of the braking system (the drums or discs) to ensure immobility. Thus, compared to actual braking when frictional heat dissipates because of rotation, now it takes longer to dissipate the heat under the surfaces in contact, especially in the case of the drum brakes, where this contact area is much larger. These explain the superior thermal behavior of the disc brakes.

**Figure 20:**
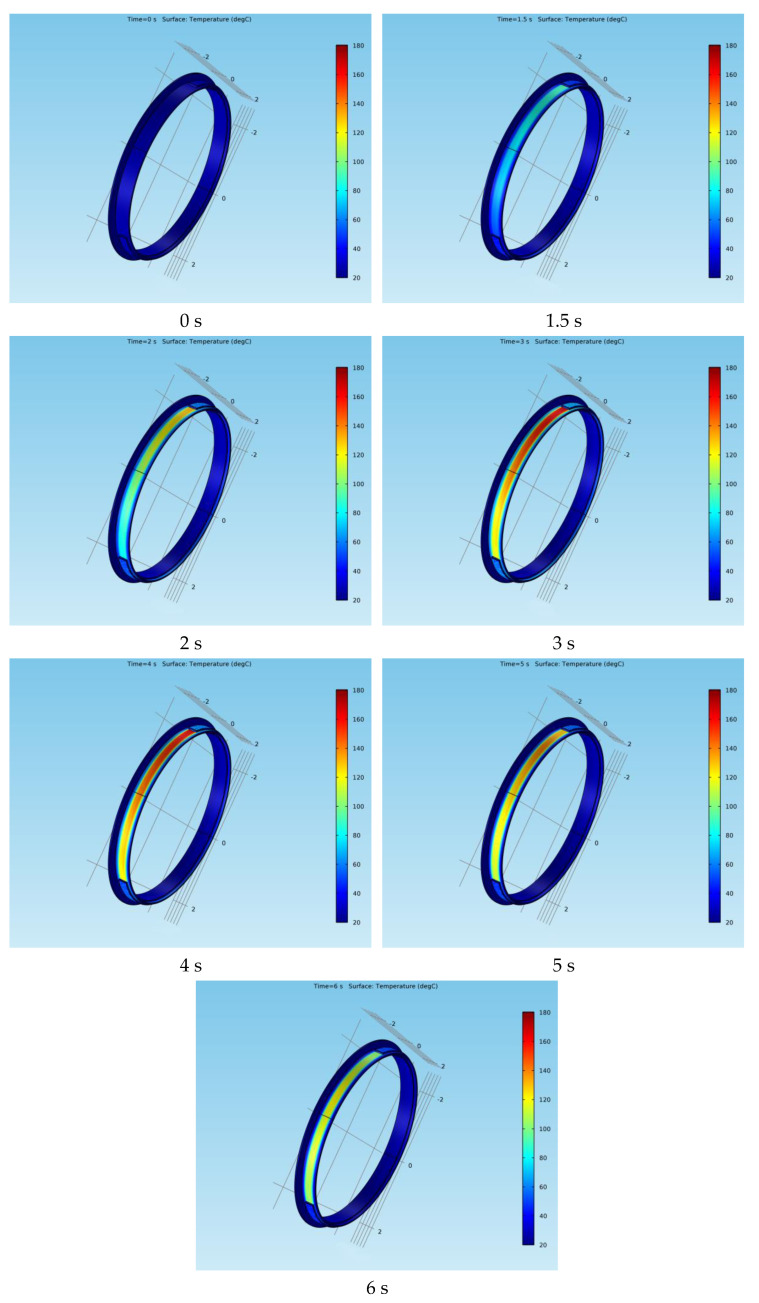
Surface temperature variation in position for the drum-and-shoe brake system.

**Figure 21:**
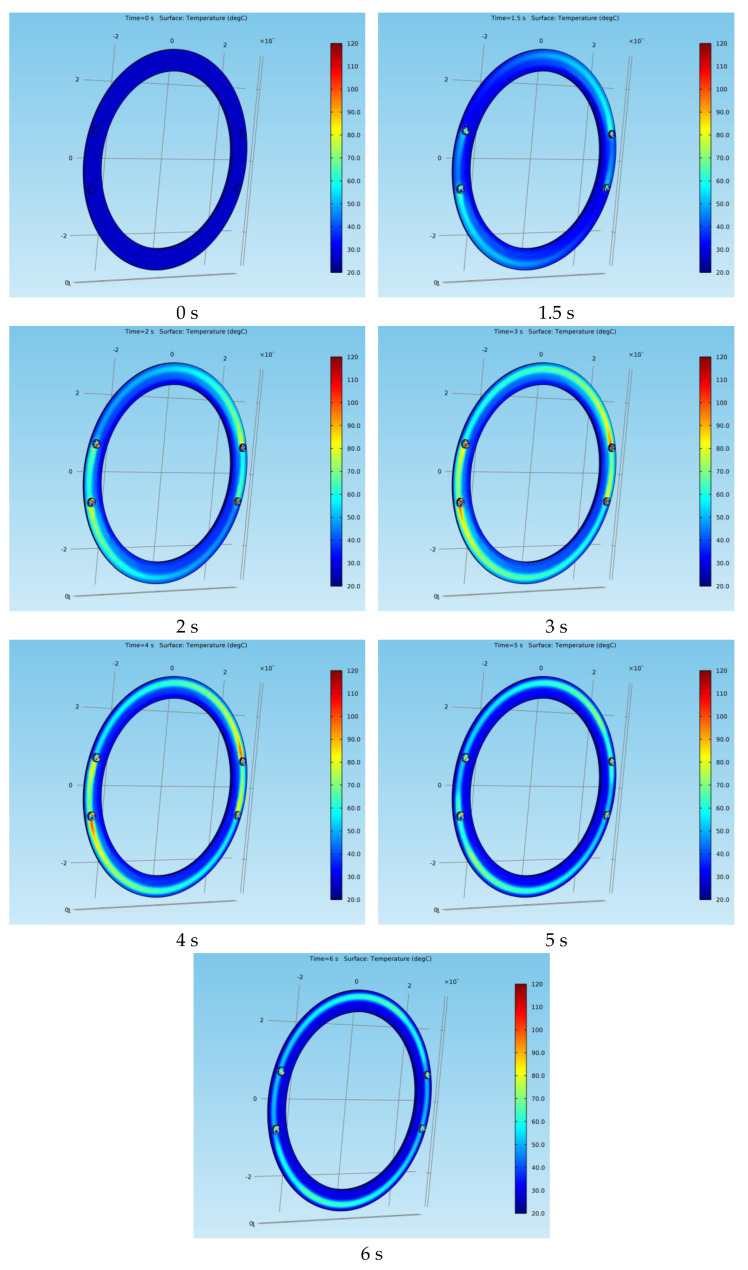
Surface temperature variation in position for the disc-and-pad brake system.

Next, the variation in the position of the surface temperature for both types of braking systems at certain moments during the emergency braking is presented. Figure 20 shows the surface temperature variation of the drum at times *t* = 0 s, 1.5 s, 2 s, 3 s, 4 s, 5 s and 6 s, while Figure 21 shows the surface temperature variation of the disc at the same time frames.

**Figure 22:**
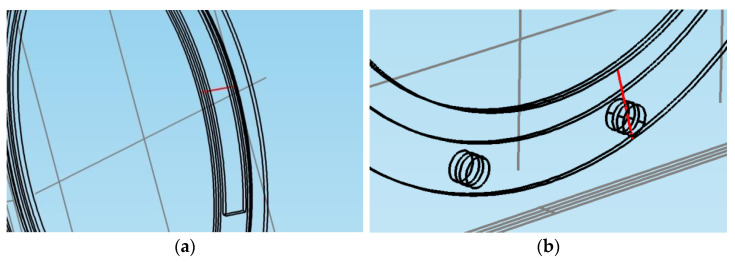
3D cut line through the fixed and rotational elements for: (**a**) drum-and-shoe brake system; (**b**) disc-and-pads brake system.

**Figure 23:**
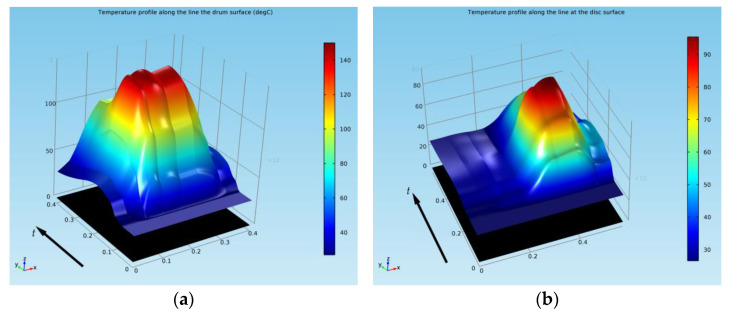
Temperature versus time profile along the 3D cut line for: (**a**) drum-and-shoe brake system; (**b**) disc-and-pads brake system.

In order to further investigate the thermal behavior of both the fixed and rotational elements in the case of both braking systems, using the *Results > Data Sets* feature of COMSOL, a 3D cut line was traced and was positioned, as shown in Figure 22, through the middle of the drum and shoe and through the middle of the disc-and-pads couple, respectively. The temperature versus time profile along this line is plotted for the drum-and-shoe brake system in Figure 23a and for the disc-and-pads brake system in Figure 23b.

**Figure 24:**
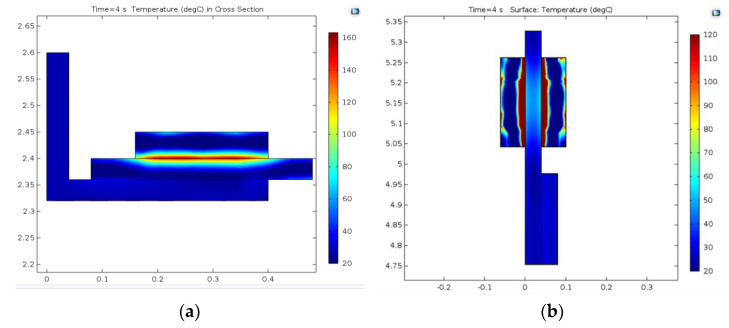
Temperature in cross-section for: (**a**) drum-and-shoe couple; (**b**) disc-and-pads couple.

The temperature at *t* = 4 s in a cross-section plane built on the cut line is shown for the drum-and-shoe couple in Figure 24a and the disc-and-pads couple in Figure 24b.

For both models, it is visible that the temperature of the active elements (the shoes and pads) exceeded the temperature of the passive elements (the drum and disc). By comparing the results in the case of the active elements only, it was found that the pad’s temperature was higher than the temperature of the shoe. This is explained by the contact surfaces for which the kinetic energy was converted to heat, approximately 6.6 times larger in the case of the shoes compared to the pads.

**Figure 25:**
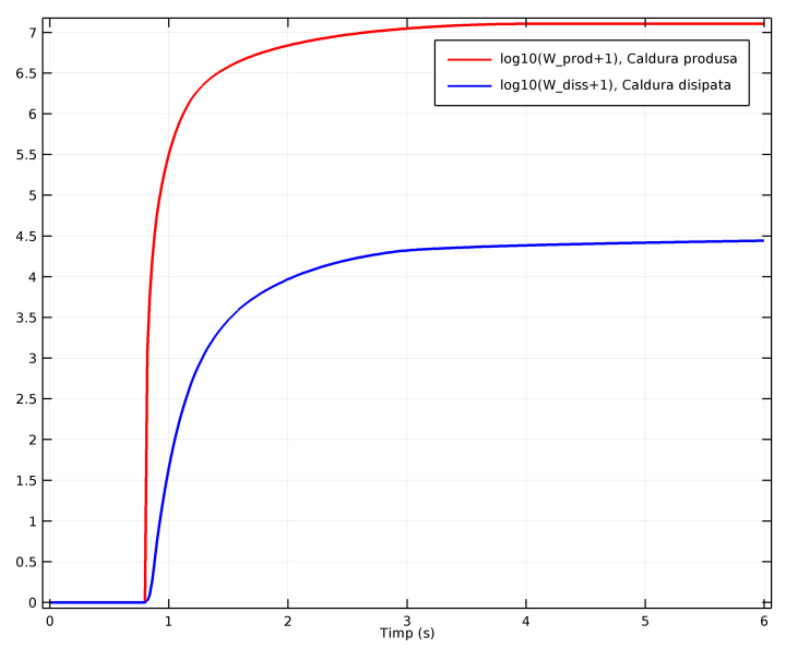
Comparison of the generated (red line) and dissipated heat (blue line).

Part of the heat generated during emergency braking gets dissipated by radiation and convection to the air, as mentioned in Section 3.3. In order to investigate how much of the generated heat was dissipated to the air, COMSOL calculated the integrals of the produced and dissipated heat as functions of time, based on the total heat rate, as represented in Figure 25. The degree of the heat dissipation in the environment by convection was proportional to the size of the dissipation surface and the speed of the rotation element (the drum or disc). The intensity of the convection heat decreased during braking because of the reduction in the free convective surface of the disc or drum, as a result of their contact with the shoes or pads. For the analyzed models, the total surface of the drums was 12.35 m^2^ and of the shoes was 4.02 m^2^, while in the case of the discs, the total surface was 38.4 m^2^, with the pads having a surface of 0.61 m^2^. During the brake contact, this meant a reduction in the free, convective surface by 32.56% in the case of the drum-and-shoe brake systems and by only 1.58% in the case of the disc-and-pads brake system.

### 5.2. Discussion of Mechanical Behavior during Emergency Braking

The heating of elements during emergency braking induces thermal stresses due to the different coefficients of the thermal expansion of materials. As a result, mechanical stresses and deformations also appear, so they were investigated. On the same COMSOL models created for each brake system and used for the thermal simulations, using the *Solid mechanics* module, a mechanical simulation was run to find the von Mises stress for the elements of both brake models at the same time *t* = 4 s. 

**Figure 26:**
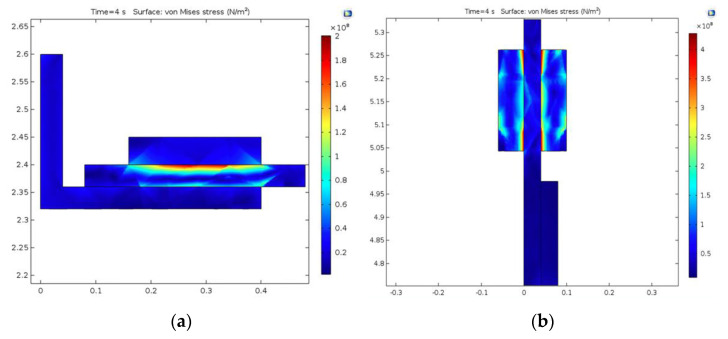
von Mises stress for: (**a**) drum-and-shoe couple; (**b**) disc-and-pads couple.

Figure 26a shows the effective stress (von Mises) for the drum-and-shoe brake couple. The highest stress occurred on the external surface of the brake drum, at contact with the shoe, with a value of 2 × 10^8^ N/m^2^, which is lower than the material yield stress of 3.2 × 10^8^ N/m^2^ for the drum (Table 1), meaning that the brake drum material was not irreversibly deformed. The von Mises stress for the disc-and-pad brake couple is shown in Figure 26b, where the maximum value of 4 × 10^8^ N/m^2^ occurred towards the edges of the brake pad at the contact surface with the disc, with values also under the yield stress of 5.5 × 10^8^ N/m^2^ for the disc (Table 1).

**Figure 27:**
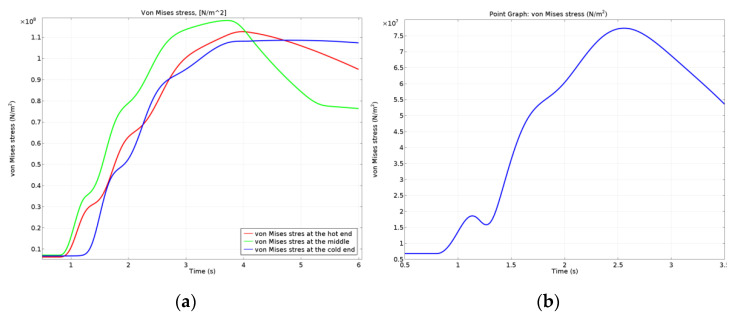
von Mises stress variation in time for: (**a**) drum; (**b**) disc.

The variation in time for the von Mises stress for the drum (corresponding to the three points investigated) is plotted in Figure 27a, and for the disc is plotted in Figure 27b. It is visible that the highest von Mises stress for the drum brake system appeared at the middle point, and for all three points investigated, the maximum values occurred before the stopping moment of the brake drum rotation. The von Mises stress in the disc brake systems was 25–30% lower than the drum brake.

**Figure 28:**
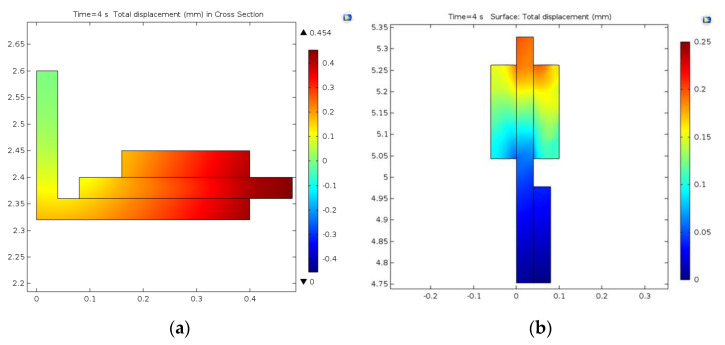
Cross-section of deformation for: (**a**) drum-and-shoe couple; (**b**) disc-and-pads couple.

Regarding the deformations of the brake elements for both models, the results are presented in cross-sections in Figure 28. In the case of the drum-and-shoe brake system, the biggest deformation in the cross-section of the brake couple appeared towards the outer boundary of the drum, with a value of 0.45 mm, as visible in Figure 28a. The deformation for the disc-and-pad brake system, also in cross-sections, is shown in Figure 28b. It had a maximum value of 0.25 mm and was situated at the external diameter of the disc towards the edge.

**Figure 29:**
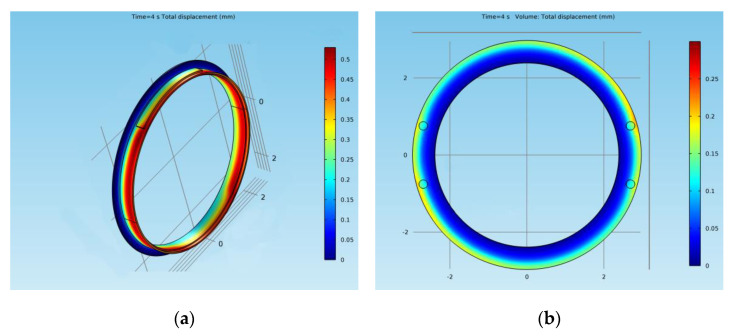
Global representation of deformation for: (**a**) drum-and-shoe couple; (**b**) disc-and-pads couple.

**Figure 30:**
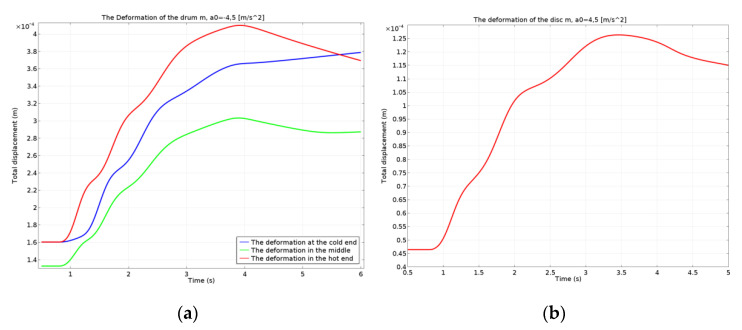
Deformation variation in time for: (**a**) drum-and-shoe system; (**b**) disc-and-pads system.

The deformations are represented globally on the complete model, in Figure 29, for both types of brake systems. The variation in the time of the deformation for the drum (corresponding to the three points investigated) is shown in Figure 30a, and for the disc in Figure 30b. For the drum brake systems, the biggest deformation occurred at the hot point of the drum and the smallest deformation occurred at the middle point, as the brake shoe did not allow the drum to deform further, explaining the maximum von Mises stress previously described. For all three points, the deformation of the drum brake system was larger than the disc brake system. 

As a limitation of this study, the lack of experimental data should be mentioned, since emergency braking tests in real mines cannot be performed, as mentioned earlier in this paper, due to security and logistic concerns.

## 6. Conclusions

During the braking process, kinetic energy transforms to frictional heat. Too high temperatures lead to a decreased efficiency of the brakes and an increased wear of the fixed elements (the shoes and pads) of the braking system. The total transmissivity of the brake elements due to their conductivity through conduction is a characteristic of the material and not the element itself. Composite friction materials used for the active elements such as the brake shoes and pads have lower conductivity than the metallic materials of the passive elements such as the drums and discs. This means that the drums and discs have a primary role in dissipating the frictional heat resulting from braking, so the study of their thermal regime is important. In the case of the mine hoists emergency braking, there is an increase in frictional heat until the speed becomes 0, with the surface temperatures reaching approximately 97 °C for the disc and 159 °C for the drum. This heat is dissipated by the convection and radiation of the brake system components. Besides the emergency braking, it must be emphasized that repetitive and successive regular braking also leads to the heating of the brake shoe friction material and can even lead to its ignition. In the case of the disc-and-pads brake systems, this shortcoming is avoided by sequentially controlling the brake disc drive systems so that optimal temperatures and the uniform wear of the pads’ linings is achieved due to periodic change in the order in which the pads come into operation.

Besides the actual temperatures resulting from emergency braking at the stopping moment, the temperature variation in time was presented. Additionally, the variation in the position of the temperature for certain moments during braking and a cross-section representation of the temperatures were discussed in order to compare the temperatures of the active and passive elements of the brake systems. It was concluded that the active components (the shoes and pads) had higher temperatures than the passive components (the drum and disc). By comparing the temperatures of the active elements only, it was found that the pad’s temperature was higher than the temperature of the shoe.

Based on the thermal analysis of the brake models in both constructive variants, the resulting von Mises stresses of the brake couples was obtained. In the case of the drum brake system, the highest stress occurred on its external surface at contact with the shoe, with a value of 2 × 10^8^ N/m^2^. The von Mises stress in the case of the disc brake system had a maximum value of 4 × 10^8^ N/m^2^, situated towards the edges of the pad at the contact surface with the disc. In both cases, these stresses were under the yield stress of the materials.

The deformations of the drum and disc were also obtained. In the case of the drum-and-shoe brake system, the maximum deformation of 0.45 mm appeared towards the outer boundary of the drum. For the disc-and-pad brake system, the maximum value of the deformation was lower than the drum, being only 0.25 mm, and it was situated at the external diameter of the disc towards the edge. 

The higher temperatures and larger deformations in the case of the drum brakes require more frequent maintenance operations, thus increasing operational costs. In the case of the drum-and-shoe system, it was concluded that the greatest deformation due to frictional heat occurred in cross-sections at the outer boundary of the drum. This lead to an uneven contact between the drum and the shoe, decreasing the braking efficiency and producing an abnormal wear of the brake shoe lining. In the case of disc-and-pads braking systems, the deformation of the discs was radial, which did not affect the uniformity of the contact between the pads and the discs, so the braking efficiency was better and the wear of the discs and pads material was more uniform along the contact surface. A possible future direction of research is the investigation of wear in the linings of active brake components. 

For every mine hoist, the transported payloads are known for every transport cycle. Additionally, the total number of transport cycles performed during a certain period of time is recorded. Based on this data, the thermal regime of the brakes during normal operation can be determined for a given period of time, and the prediction of material failure for the brake system components can be achieved using computer simulations. This is another possible future research direction. COMSOL Multiphysics allows extensive thermo-mechanical simulations for various configurations of brakes from the dimensions, number of elements and material characteristics point of view.

## Figures and Tables

**Table 1:** Material properties for the brake elements.

Property	Unit	Drive Wheel	Disc	Pad	Drum	Shoe
Thermal Conductivity	W/(m∙K)	44.5	76.2	8.7	50	8.7
Density	kg/m^3^	7850	7870	2000	7000	2000
Surface emissivity		0.28	0.2	0.8	0.28	0.8
Yield Strength	N/m^2^	6.2 × 10^8^	5.5 × 10^8^	8.63 × 10^7^	3.5 × 10^8^	2.93 × 10^8^
Specific Heat	J/(kg∙K)	475	440	935	420	935
Poisson’s Ratio		0.33	0.29	0.25	0.25	0.25
Young’s Modulus	Pa	200 × 10^9^	200 × 10^9^	140 × 10^9^	140 × 10^9^	140 × 10^9^
Thermal Expansion Coefficient	1/K	12.3 × 10^−6^	12.3 × 10^−6^	12.3 × 10^−6^	11 × 10^−6^	12.3 × 10^−6^
Material		Alloy steel	Cast Iron	Friction material	Steel	Friction material

**Table 2:** Values and parameters for *Heat Flux*.

Brake Model Type	Heat Transfer Coefficient		Plate Length (m)	Velocity, Fluid (m/s)	Fluid	Absolute Pressure (Pa)	External Temp. (K)
Drum-and-shoe	External forced convection	Plate, Averaged transfer coefficient	2.4	v(t)	Air	1 [atm]	T_air
Disc-and-pads	External forced convection	Plate, Averaged transfer coefficient	2.78	v(t)	Air	1 [atm]	T_air

**Table 3:** Characteristics, values and options for *Thermal Contact*.

Characteristic	Option	Value
Constriction conductance	Cooper–Mikic–Yovanovich correlation	
Gap conductance (W/m^2^∙K)	User defined	0
Surface roughness, asperities average heigh (m)		1 [um]
Surface roughness, asperities average slope		0.4
Contact pressure (N/m^2^)	User defined	ht.tc1.Qb/(mu*v(t))
Hardness definition (Pa)	Microhardness	800 [MPa]
Heat partition coefficient	Charron’s relation	
Overall heat transfer rate		-m_car*v(t)*a(t)/2

**Table 4:** *Global equations* for the two brake systems.

Name	f(u, ut, utt, t) (W)	Initial Value (u_0) (J)	Initial Value (u_t0) (W)	Description
W_prod	W_prod W_prodt-intop1(ht.tc1.Qb)	0	0	Produced heat
W_diss	W_diss W_disst+(intop2(ht.q0+ht.rflux))	0	0	Dissipated heat

**Table 5:** *Linear Elastic material* for the two brake systems.

Characteristic	Option
Solid model	Isotropic
Specify	Young’s modulus and Poisson’s ratio
Young’s modulus	From material
Poisson’s ratio	From material
Density	From material

## Data Availability

Not applicable.
